# The GhSWEET42 Glucose Transporter Participates in *Verticillium dahliae* Infection in Cotton

**DOI:** 10.3389/fpls.2021.690754

**Published:** 2021-07-27

**Authors:** Mengxi Sun, Zhiqiang Zhang, Zhongying Ren, Xingxing Wang, Wenjie Sun, Hongjie Feng, Junjie Zhao, Fei Zhang, Wei Li, Xiongfeng Ma, Daigang Yang

**Affiliations:** ^1^State Key Laboratory of Cotton Biology, Key Laboratory of Biological and Genetic Breeding of Cotton, Ministry of Agriculture and Rural Affairs, Institute of Cotton Research, Chinese Academy of Agricultural Sciences, Anyang, China; ^2^Zhengzhou Research Base, State Key Laboratory of Cotton Biology, Zhengzhou University, Zhengzhou, China; ^3^Department of Plant Science, School of Agriculture and Biology, Shanghai Jiao Tong University, Shanghai, China

**Keywords:** cotton, SWEET, *Verticillium dahliae*, glucose, sugar transporter

## Abstract

The SWEET (sugars will eventually be exported transporter) proteins, a family of sugar transporters, mediate sugar diffusion across cell membranes. Pathogenic fungi can acquire sugars from plant cells to satisfy their nutritional demands for growth and infection by exploiting plant SWEET sugar transporters. However, the mechanism underlying the sugar allocation in cotton plants infected by *Verticillium dahliae*, the causative agent of *Verticillium* wilt, remains unclear. In this study, observations of the colonization of cotton roots by *V. dahliae* revealed that a large number of conidia had germinated at 48-hour post-inoculation (hpi) and massive hyphae had appeared at 96 hpi. The glucose content in the infected roots was significantly increased at 48 hpi. On the basis of an evolutionary analysis, an association analysis, and qRT-PCR assays, *GhSWEET42* was found to be closely associated with *V. dahliae* infection in cotton. Furthermore, *GhSWEET42* was shown to encode a glucose transporter localized to the plasma membrane. The overexpression of *GhSWEET42* in *Arabidopsis thaliana* plants led to increased glucose content, and compromised their resistance to *V. dahliae*. In contrast, knockdown of *GhSWEET42* expression in cotton plants by virus-induced gene silencing (VIGS) led to a decrease in glucose content, and enhanced their resistance to *V. dahliae*. Together, these results suggest that *GhSWEET42* plays a key role in *V. dahliae* infection in cotton through glucose translocation, and that manipulation of *GhSWEET42* expression to control the glucose level at the infected site is a useful method for inhibiting *V. dahliae* infection.

## Introduction

*Verticillium dahliae* is a soil-borne fungal pathogen that can infect a broad range of plant species, including many economically important crops, and causes devastating diseases (Fradin and Thomma, 2006). During infection, *V. dahliae* hyphae pass through the epidermal cells and multiply in the vascular tissue (Zhao et al., 2014). The hyphae then produce toxins and block plant ducts, thereby disrupting water transport, and ultimately leading to plant death (Fradin and Thomma, 2006). All microorganisms that interact with plants must obtain metabolites from the host cells to meet the nutrient requirements for growth (Yamada et al., 2016). Since carbon is essential for growth and pathogenicity, obtaining sufficient sugars from plants is an important task for successful infection by plant pathogens (Chen et al., 2010; Klimes et al., 2015). However, little is known about the molecular mechanism of sugar uptake by *V. dahliae* from plants.

During long-term co-evolution, plants and microorganisms have developed a set of strategies enabling them to compete for nutrients (Chen et al., 2012). Plant hosts can prevent metabolite loss to pathogens by redistributing sugars away from the infection site (Berger et al., 2004). In *Arabidopsis thaliana*, the sugar transport protein AtSTP13 was activated by phosphorylation during bacterial challenge, which enhances its hexose uptake activity to compete with bacteria for extracellular sugars (Yamada et al., 2016). However, pathogens have evolved mechanisms that increase sugar concentrations at the infection site by modulating host sugar transporters, including SWEETs (sugars will eventually be exported transporters) (Cox et al., 2017). Both fungal and bacterial pathogens can induce the expression of different SWEET genes, suggesting that the sugar transport function of SWEETs may be targeted by pathogens and symbionts for nutrient acquisition (Chen et al., 2010). For example, in maize, *Ustilago maydis* infection was found to affect the local expression of the sugar transporter genes *ZmSWEET4a* and *ZmSWEET4b*, with expression levels correlated with fungal biomass (Sosso et al., 2019). In a previous study, *Rhizoctonia solani* upregulated *OsSWEET11* expression 5- and 3-fold in rice leaves and sheaths, respectively, at 72 h after infection (Gao et al., 2018). Recent studies have shown that blocking pathogen access to host sugars resulting in its sugar starvation is a promising strategy for controlling plant diseases. For example, silencing the hexose transporter gene *PsHXT1* restricted the normal growth and development of *Puccinia striiformis* f. sp. *Tritici* (*Pst*), leading to decreased fungal biomass and reduced symptoms of wheat stripe rust disease (Chang et al., 2020). In addition, the expression up-regulation of the wheat sugar transporter gene *TaSTP6* contributes to the acquisition of host sugars by *Pst*, and knock-down of *TaSTP6* reduced the susceptibility of wheat to *Pst* (Huai et al., 2019). The inhibition of *OsSWEET11* function in leaf cells mitigated the symptoms of sheath blight disease in rice plants (Gao et al., 2018).

Sucrose is the major sugar translocated in most plants, and it is likely to be the type of sugar used by plant pathogenic fungi (Bezrutczyk et al., 2018). However, some studies have indicated that glucose, not sucrose, is the main carbon source obtained from the host by fungal mycelia (Sutton and Hall, 1999). A previous study found that, during infection of plants by *U. maydis*, the glucose content in the infected plant part was 20-times that in the uninfected part at 8 day post-infection (Doehlemann et al., 2008). Analyses of sugar acquisition and metabolism by *U. maydis* revealed a glucose concentration gradient in the hyphae, with a high concentration at the highly active hyphal tip (Sosso et al., 2019). Additionally, Hxt1 is a high-affinity glucose, fructose, and mannose transporter in *U. maydis*, and its deficient mutant strains showed significantly reduced growth in, and pathogenicity toward, their hosts (Schuler et al., 2015).

In this study, observations of the *V. dahliae* colonization process revealed that *V. dahliae* hyphae had penetrated into the roots of cotton (*Gossypium hirsutum* L.) plants at 48-hour post-inoculation (hpi). The glucose content in the infected roots had also significantly increased at 48 hpi. The transcript level of *GhSWEET42*, which encodes a plasma membrane-localized glucose transporter, was significantly induced at 48 hpi in the infected roots. Overexpression of *GhSWEET42* in *A. thaliana* increased the glucose content in the transgenic plants, and made them more susceptible to *V. dahlia* infection. Conversely, knockdown of *GhSWEET42* in cotton plants resulted in decreased glucose content, leading to improved resistance to *V. dahliae*. Our findings suggest that *GhSWEET42* is involved in *V. dahliae* infection through glucose translocation.

## Materials and Methods

### Plant Materials and Growth Conditions

All *A. thaliana* lines used in this study were in the Columbia (Col-0) genetic background. Seeds were surface-sterilized with 0.1% mercuric chloride before being sown on the surface of the agar culture medium. Then seeds were vernalized at 4°C for 2 day in darkness and transferred to a growth chamber for germination. The growth chamber was set at 22°C and 60% relative humidity, with a 16-h light/8-h dark cycle.

The cotton cultivar Zhongzhimian 2, which is resistant to *V. dahliae*, was used in this study. Seeds were placed in sterile water and kept in an incubator at 30°C to germinate overnight. Seeds with undamaged radicles of the same length were selected and rinsed with water. Each seed was sown with the radicle pointing downwards in nutrient medium. Cotton seedlings were grown in a growth chamber set at 26°C and 60% relative humidity with a 16-h light/8-h dark cycle.

### *V. dahliae* Growth Conditions and Plant Inoculations

The *V. dahliae* strain V592 was grown on potato dextrose agar medium for 4 day, after which it was transferred to Czapek’s medium [0.3% (w/v) NaNO_3_, 0.1% (w/v) MgSO_4_, 0.1% (w/v) KH_2_PO_4_, 0.0002% (w/v) FeSO_4_, 0.1% (w/v) KCl, and 3% (w/v) sucrose, pH 6.0] and incubated at 25°C for 5 day (Gao et al., 2013).

The *A. thaliana* inoculations were performed by root dipping, as follows: the roots of 4-week-old seedlings were rinsed with water and then immersed in a *V. dahliae* conidial suspension (1 × 10^7^ conidia/mL) for 90 s, after which the seedlings were replanted in fresh soil. For cotton inoculations, cotton plants were grown in pots in a growth chamber for 1 month. Conidial suspensions were diluted with distilled water to a final concentration of about 1 × 10^7^ conidia/mL. A 10-mL aliquot of the conidial suspension was added to each pot using a syringe to infect cotton seedlings (Gong et al., 2018).

### Disease Assessment After *V. dahliae* Inoculation

To quantify the fungal biomass in plants, genomic DNA was extracted from various tissues collected after inoculating plants with V592 for quantitative real-time PCR (qRT-PCR) analysis. The internal transcribed spacer (ITS) region of ribosomal DNA was targeted using the fungal-specific ITS1-F primer and the *V. dahliae*-specific reverse primer STVe1-R (Fradin et al., 2011). The *A. thaliana AtUBQ5* gene was used as an endogenous plant control (Gutierrez et al., 2008). The qRT-PCR analyses were completed using the extracted genomic DNA as previously described (Santhanam et al., 2013).

In the *V. dahliae* infection recovery assay, 1-cm sections cut from the base of the stem were surface-sterilized in 70% ethanol and rinsed with sterile water at 2 week after infection by V592. The stem segments were placed on potato dextrose agar medium supplemented with cephalosporin and incubated for 3 day at 25°C before being examined and photographed (Gong et al., 2018).

The disease symptom severity was recorded using an index ranging from 0 (healthy plants) to 4 (dead plants) (Zhang et al., 2017). The disease index was calculated using the formula described in the previous study (Gong et al., 2018).

### Confocal Observation of *V. dahliae* Infection Process in Cotton Roots

Seeds of the cotton cultivar Zhongzhimian 2 were placed in a seed germination bag. After 5 day, cotton seedlings with uniform size were immersed in a V592-GFP conidial suspension (1 × 10^7^ conidia/ML) for 90 s. The roots were cut lengthwise at 0, 6, 12, 24, 48, and 96 hpi and then examined under a confocal laser scanning microscope.

### Phylogenetic and Association Analysis of *SWEET* Genes in Cotton

The protein sequences of SWEET genes in *G. hirsutum*, *A. thaliana*, and *Vitis vinifera* (Supplementary Table 1) were obtained from the literatures (Chen et al., 2010; Chong et al., 2014; Li et al., 2018). Multiple sequence alignments were performed using Clustal X (version 2.0) (Larkin et al., 2007). Phylogenetic trees were constructed using the neighbor-joining method with 1000 bootstrap replicates in MEGA (version 7.0) (Kumar et al., 2016).

Data related to the *V. dahliae* resistance of 258 modern *G. hirsutum* cultivars and enhanced germplasm lines were obtained from a previous study (Fang et al., 2017). The single nucleotide polymorphisms (SNPs) located in the clade II SWEET genes from *G. hirsutum* were identified and extracted. A general linear model (GLM), a GLM model with principle component analysis (GLM + PCA), and a mixed linear model (MLM) were used to perform association analyses between these SNPs and Verticillium wilt resistance using TASSEL (version 5.0) (Mao et al., 2020).

### Ectopic Expression of *GhSWEET42* in *A. thaliana*

The full-length *GhSWEET42* coding sequence was cloned using gene-specific primers (Supplementary Table 2). The cloned sequence was inserted into a modified pCAMBIA2300 plant binary vector *via* homologous recombination. A freeze-thaw method was used to transform *Agrobacterium tumefaciens* strain GV3101 with the recombinant vector. The floral-dip method (Clough and Bent, 2010) was used to generate transgenic *A. thaliana* plants, which were selected on Murashige and Skoog medium containing kanamycin. Transgenic *A. thaliana* plants from the T_3_ generation were used for follow-up experiments.

### Virus-Induced Gene Silencing (VIGS) of *GhSWEET42* in Cotton

The tobacco rattle virus (TRV) system was used for a VIGS analysis in cotton as previously described (Wang et al., 2014). A 250-bp *GhSWEET42* fragment was inserted into the pTRV2 vector *via* homologous recombination to generate the *TRV:GhSWEET42* construct. Similarly, the *TRV:GhCLA1* construct was produced as a positive control to monitor gene silencing efficiency. The empty vector (*TRV:00*) was used as a negative control. All vectors were inserted into *A. tumefaciens* strain GV3101 cells, which were then injected into the cotyledons of 7-day-old cotton seedlings (Long et al., 2019).

### Total RNA Extraction, RT-PCR, and qRT-PCR

Total RNA was isolated using the RNAprep Pure Plant Kit (Tiangen, Beijing, China). First-strand cDNA was synthesized using the PrimeScript^TM^ II 1st Strand cDNA Synthesis Kit (Takara, Dalian, China). The RT-PCR assays were conducted as described in a previous study (Bustin, 2000). The qRT-PCR assays were performed using the Light Cycler 480 system and the SYBR Premix Ex Taq Kit (Takara). The *G. hirsutum GhHIS3* and *A. thaliana AtUBQ5* genes were used as internal controls. The primers used in this experiment are listed in Supplementary Table 2.

### Subcellular Localization of *GhSWEET42*

Cells of *A. tumefaciens* GV3101 were transformed with the pCAMBIA2300 vector carrying *GhSWEET42* under the control of the CaMV 35S promoter to produce *GhSWEET42* with a C-terminal green fluorescent protein (GFP) tag. The transformed GV3101 cells were cultured and used to infiltrate leaves of tobacco (*Nicotiana benthamiana*) as previously described (Sosso et al., 2015). Bacterial cultures were grown for 16 h, after which the cells were collected by centrifugation and resuspended in infiltration buffer (10 mM MES, pH 5.6, 10 mM MgCl_2_, and 200 μM acetosyringone) to an optical density at 600 nm (OD_600_) of approximately 1.0–1.2. The abaxial side of *N. benthamiana* leaves was infiltrated with the cell solution using a needleless syringe. The infiltrated plants were incubated for 24 h, and then green fluorescence was visualized under a confocal laser scanning microscope.

Protoplasts were isolated from the young leaves of *A. thaliana* seedlings as previously described (Yoo et al., 2007). The protoplasts were transiently transformed by adding 10 μg vector to a 100-μL protoplast solution (10^5^ protoplasts/mL), which was then gently mixed and incubated at room temperature for 12 h. Green fluorescence was detected by confocal laser scanning microscopy.

### Yeast Complementation Assays

The *GhSWEET42* coding sequence was cloned into the pDR196 expression vector. The sucrose transport-deficient *S. cerevisiae* mutant *SUSY7/ura3* (Riesmeier et al., 1993) was transformed with the pDR196-*GhSWEET42* recombinant vector and then grown on selective synthetic complete medium (without uracil) containing 2% glucose as the sole carbon source. Drop tests were used to assess the growth of the transformed yeast on agar-solidified SD medium supplemented with 2% sucrose (OD_600_ = 1; 1:10, 1:100, 1:1,000, and 1:10,000 dilutions). Yeast cells transformed with pDR196-*AtSUT4* served as the positive control, and cells carrying the empty vector were used as the negative control. The transformed yeast cells were grown at 30°C for 4 day. Glucose-supplemented medium was used as the positive growth medium.

The hexose transport-deficient *Saccharomyces cerevisiae* mutant EBY.VW4000 (Wieczorke et al., 1999) was transformed with pDR196-*GhSWEET42*, pDR196-*HXT7* (positive control), or pDR196 (negative control) for complementation assays. Drop tests were used to assess the growth of the transformed yeast on agar-solidified SD medium supplemented with 2% glucose or fructose (OD_600_ = 1; 1:10, 1:100, 1:1,000, and 1:10,000 dilutions). Yeast cells were grown at 30°C for 4 days. Maltose -supplemented medium was used as the positive growth medium. Specifically, the yeast sugar transport-defective strains *SUSY7/ura3* and EBY.VW4000 have different growth states on media with glucose as the sole carbon source.

### Determination of Glucose Contents

Each plant sample (0.2 g) was ground to a powder and then 500 μL of a mixture of methanol, isopropanol, and water (3:3:2, V/V/V) was added. The mixture was vortexed for 3 min, sonicated for 30 min, and then centrifuged at 14,000 rpm for 3 min at 4°C. The supernatant was collected and mixed with the internal standard and then evaporated under a stream of nitrogen. The evaporated samples were freeze-dried, and then each dried sample was derivatized with 100 μL pyridine solution of methoxylamine hydrochloride (15 mg/mL) at 37°C for 2 h. The mixture was added to 100 μL BSTFA (with 1% TMSC) and kept at 37°C for 30 min after vortexing. All mixtures were analyzed by as chromatography-mass spectrometry (GC-MS) as previously described (Gómez-González et al., 2010; Zheng et al., 2016).

## Results

### Colonization of Cotton Roots by *V. dahliae* and Subsequent Changes in Glucose Content

**FIGURE 1:**
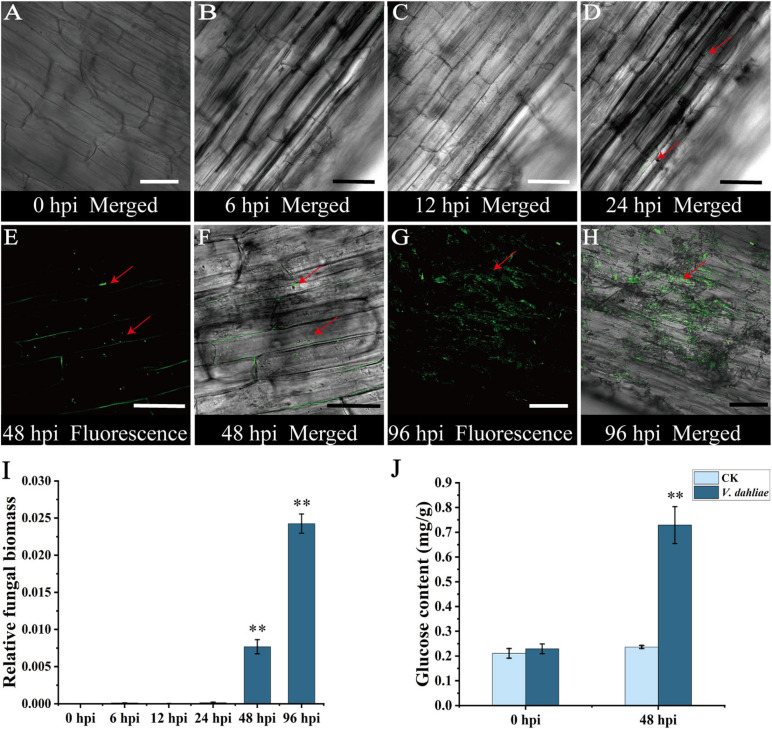
Confocal micrographs of initial stage of cotton root colonization by *Verticillium dahliae* and post-infection changes in glucose content. **(A)** Longitudinal section of cotton roots with no detectable fluorescence at 0 hours post-inoculation (hpi). **(B)** Longitudinal section of cotton roots with no detectable fluorescence at 6 hpi. **(C)** Longitudinal section of cotton roots with no detectable fluorescence at 12 hpi. **(D)** Fluorescence at the surface of cotton roots at 24 hpi. **(E)** Longitudinal section of cotton roots at 48 hpi with hyphae multiplying in the intercellular space before penetrating the cell wall and invading root cells. Red arrow indicates the hyphal position. **(F)** Merged bright field transmission image and corresponding fluorescence image at 48 hpi. Red arrow indicates the hyphal position. **(G)** Longitudinal section of cotton roots at 96 hpi with hyphae covering the entire duct tissue and spreading within it. Red arrow indicates the hyphal position. **(H)** Merged bright field transmission image and corresponding fluorescence image at 96 hpi. Red arrow indicates the hyphal position. **(I)** qRT-PCR analysis of fungal biomass at various stages of infection. Asterisks indicate significant differences compared with 0 hpi. **(J)** Changes in the glucose content in cotton roots after *V. dahliae* infection. Asterisks indicate significant differences compared with CK. Error bars represent standard deviation of three biological replicates. Data were analyzed using Student’s *t*-test (^∗∗^*P* < 0.01). Scale bar = 50 μm.

To clarify the process of colonization, cotton plants were inoculated with *V. dahliae* strain V592-GFP. The inoculated roots were cut longitudinally at various time-points and examined under a confocal laser scanning microscope. From 0 to 12 hpi, no obvious green fluorescence was detected in the cotton roots, implying that *V. dahliae* did not infect the roots before 12 hpi (Figures 1A–C and Supplementary Figure 1). Green fluorescence was first detected at 24 hpi, and the signal intensity increased significantly after 48 hpi (Figures 1D–F and Supplementary Figure 1). At 96 hpi, the green fluorescence from hyphae was observed in the longitudinal sections of cotton roots (Figures 1G,H and Supplementary Figure 2). To verify these results, the relative fungal biomass at each infection stage was determined by qRT-PCR. The *V. dahliae* fungal material was detectable at 24 hpi, and the biomass was significantly increased at 48 and 96 hpi, consistent with the fluorescence results (Figure 1I).

Glucose is a nutrient as well as a signaling molecule that affects diverse processes (Sami et al., 2019; Yoon et al., 2021). Therefore, the glucose contents of cotton roots at 0 and 48 hpi were quantified using GC-MS technology. The data indicated the glucose content had increased significantly at 48 hpi in the infected cotton root (Figure 1J), suggesting that glucose might be involved in the infection of cotton roots by *V. dahliae*.

### *GhSWEET42* Is a Clade II SWEET Sugar Transporter Related to *V. dahliae* Resistance

**FIGURE 2:**
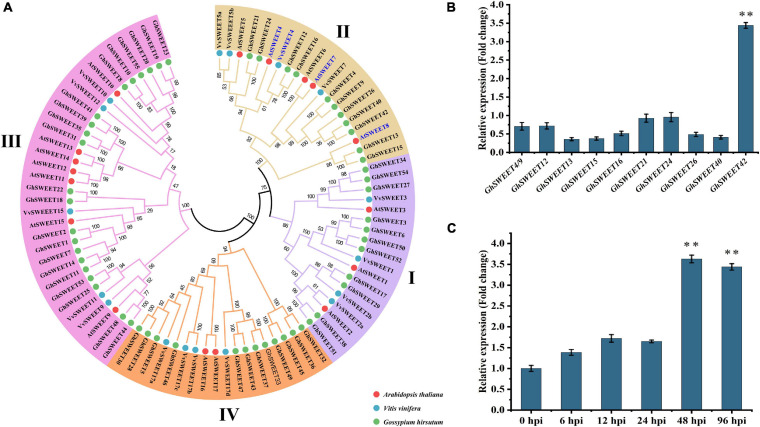
Phylogenetic and transcriptional analyses of *Gossypium hirsutum SWEET* genes. **(A)** Phylogenetic analysis of *SWEET* genes. Phylogenetic tree was constructed using protein sequences of *SWEET* genes from *G. hirsutum* (*GhSWEETs*), *Arabidopsis thaliana* (*AtSWEETs*), and *Vitis vinifera* (*VvSWEETs*). Blue mark indicates *SWEET* genes related to glucose transport and disease resistance in *A. thaliana* and *V. vinifera*. **(B)** Relative transcript levels of *G. hirsutum* clade II *SWEET* genes in roots at 48 h after infection by *Verticillium dahliae*. **(C)** Relative transcript levels of *GhSWEET42* in roots infected with *V. dahliae*. For each gene, transcript level in control roots (wounded roots treated with 10 mL water) was set to 1 to determine relative transcript levels in inoculated roots [wounded roots treated with 10 mL V592 conidial suspension (1 × 10^7^ conidia/mL)]. Asterisks represent significant difference in fold change. Error bars represent standard deviation of three biological replicates. Data were analyzed using Student’s *t*-test (^∗∗^*P* < 0.01).

Plant pathogens usually hijack plant SWEET sugar transporters to obtain sufficient energy to successfully invade and grow in host plants (Cox et al., 2017; Phillips et al., 2017; Gupta, 2020). To explore why *V. dahliae* infection leads to increased glucose contents in cotton roots, a phylogenetic analysis was completed using the previously identified *G. hirsutum SWEET* genes as well as those from *A. thaliana* and *V. vinifera* (Figure 2A). Many *SWEET* genes related to glucose transport and disease resistance in *A. thaliana* and *V. vinifera* (Chong et al., 2014) clustered in clade II of the *SWEET* gene family. Therefore, we focused our research on the *G. hirsutum SWEET* genes in this clade.

To investigate the association between the *G. hirsutum* clade II *SWEET* genes and *V. dahliae* resistance, a candidate gene association analysis was conducted to determine whether the genetic variation in the *G. hirsutum* clade II *SWEET* genes was associated with phenotypic differences in responses to *V. dahliae* among cotton varieties. Earlier research indicated that the 200-kb regions upstream and downstream of significant SNPs can be defined as quantitative trait loci in *G. hirsutum* (Sun et al., 2017; Song et al., 2019). Therefore, SNP markers within a 400-kb interval centered on each *SWEET* gene were selected from 1,871,401 high-quality SNPs (major allele frequency > 0.05) identified in a previous study (Fang et al., 2017). The *V. dahliae* resistance phenotype data for the corresponding cotton varieties were also obtained from that study (Fang et al., 2017). Three statistical models, namely a GLM, a GLM + PCA, and an MLM were used to identify significant genotypic and phenotypic associations. The results of those analyses indicated that genetic variations within or near the *G. hirsutum SWEET* genes, including *GhSWEET9*, *GhSWEET15/16*, *GhSWEET21*, *GhSWEET40*, and *GhSWEET42*, are significantly associated with *V. dahliae* resistance (Supplementary Table 3).

To elucidate the potential role of individual *G. hirsutum SWEET* genes during plant responses to a *V. dahliae* infection, their transcript levels in cotton roots at 48 hpi were examined by qRT-PCR. Of the analyzed genes, only *GhSWEET42* was strongly induced at 48 hpi. In contrast, the transcript levels of the other *G. hirsutum* clade II *SWEET* genes were unaffected by *V. dahliae* infection (Figure 2B). Additionally, *GhSWEET42* more highly expressed in the susceptible cotton variety than in the resistant cotton varieties (Supplementary Figure 3). Monitoring of the dynamic changes in *GhSWEET42* expression revealed high transcript levels of *GhSWEET42* at 48 and 96 hpi (Figure 2C). This was consistent with the colonization process and changes in glucose content following inoculation with *V. dahliae*. Accordingly, *GhSWEET42* may be important for mediating glucose translocation during *V. dahliae* infection of cotton.

### *GhSWEET42* Encodes a Plasma Membrane-Localized Glucose Transporter

**FIGURE 3:**
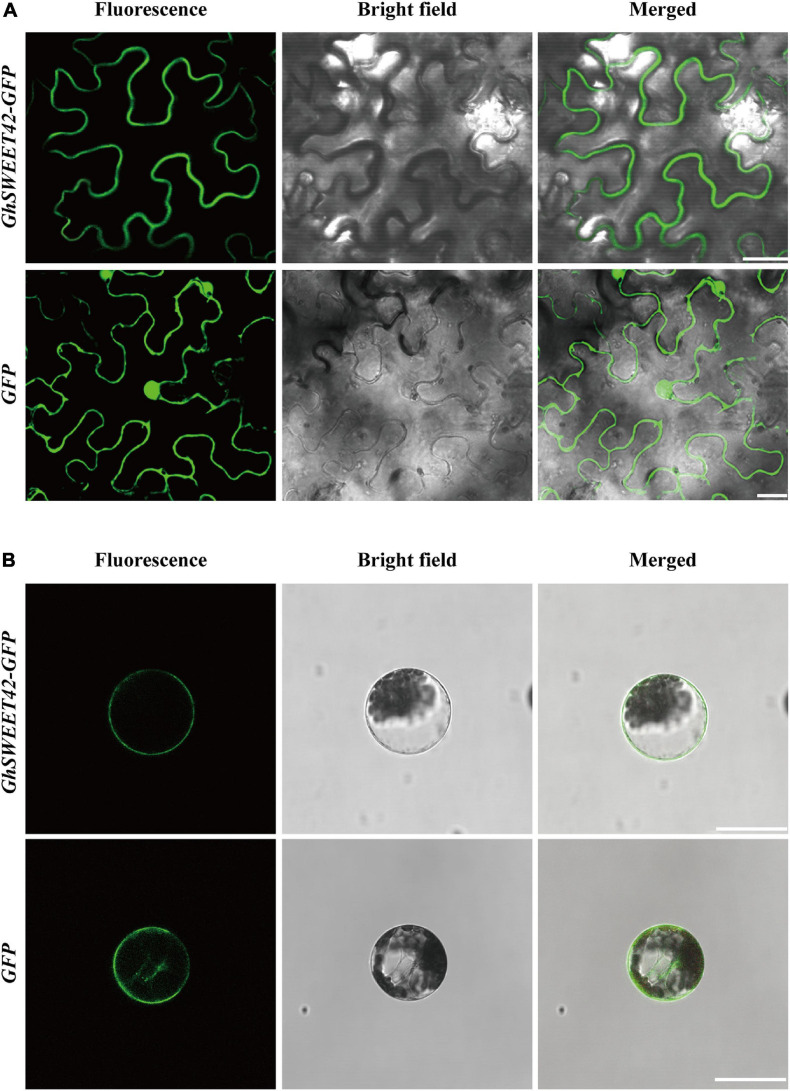
Subcellular localization of GhSWEET42. **(A)** Subcellular localization of GhSWEET42 in tobacco epidermal cells. **(B)** Subcellular localization of GhSWEET42 in *Arabidopsis thaliana* protoplasts. Scale bar = 25 μm. Plasmid containing 35S-GhSWEET42-GFP fusion (GhSWEET42-GFP) and 35S-GFP (GFP) was introduced into tobacco epidermal cells and *A. thaliana* protoplasts. GhSWEET42-GFP fusion protein localized in the plasma membrane.

On the basis of a multiple sequence alignment and transmembrane structural analysis (Supplementary Figure 4), the GhSWEET42 amino acid sequence was predicted to include seven transmembrane domains, which are conserved among SWEET proteins (Chen et al., 2010). To confirm the subcellular localization of GhSWEET42, the gene encoding the GhSWEET42-GFP fusion protein was expressed under the control of the CaMV 35S promoter in transiently transformed *N. benthamiana* epidermal cells and *A. thaliana* protoplasts. Confocal microscopy images of the GFP signal confirmed that GhSWEET42-GFP is localized in the plasma membrane (Figures 3A,B).

**FIGURE 4:**
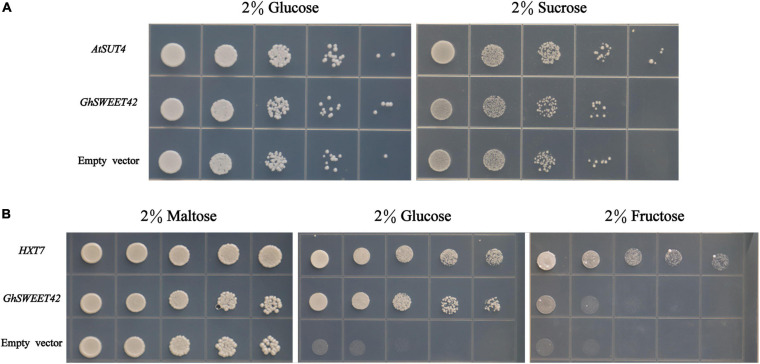
Functional characterization of GhSWEET42 using yeast sugar transport-defective strains *SUSY7/ura3* and EBY.VW4000. **(A)** Complementation of sucrose transport deficiency in the *SUSY7/ura3* yeast mutant by *GhSWEET42* (positive control: *AtSUT4*; negative control: empty vector). **(B)** Complementation of hexose transport deficiency in the EBY.VW4000 yeast mutant by *GhSWEET42* (positive control: yeast *HXT7*; negative control: empty vector).

Previous research confirmed that SWEET proteins mediate the transport of sucrose, glucose, and fructose (Sosso et al., 2015; Sun et al., 2019). To identify which sugar is transported by GhSWEET42, the sucrose transport-deficient yeast mutant *SUSY7/ura3* (Riesmeier et al., 1993) was transformed with *GhSWEET42*. The growth of the *GhSWEET42*-expressing yeast cells on medium supplemented with sucrose was similar to that of yeast cells carrying the empty vector (Figure 4A), indicating that *GhSWEET42* does not transport sucrose. The expression of *GhSWEET42* in the hexose transport-deficient yeast mutant EBY.VW4000 (Wieczorke et al., 1999) was unable to restore the growth of the yeast mutant on medium supplemented with fructose. However, *GhSWEET42* expression enabled the yeast mutant to grow on medium supplemented with glucose, similar to the positive control expressing the yeast carbohydrate transporter gene *HXT7* (Figure 4B), implying that GhSWEET42 is a glucose transporter.

### Overexpression of *GhSWEET42* in *A. thaliana* Increases Glucose Content and Weakens Resistance to *V. dahliae*

**FIGURE 5:**
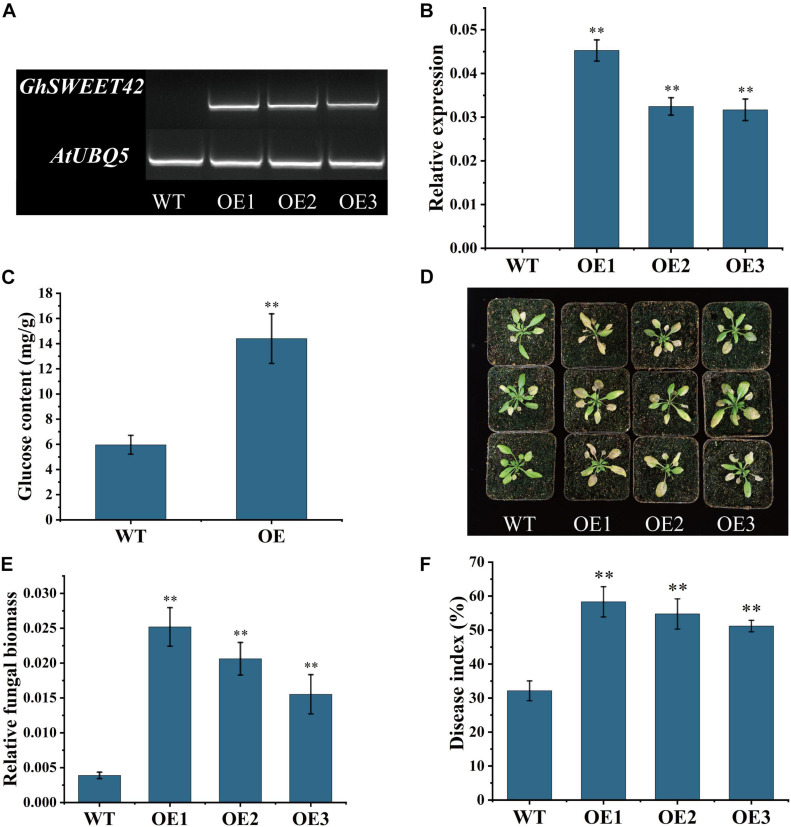
Decreased *Verticillium dahliae* resistance of *Arabidopsis thaliana* plants overexpressing *GhSWEET42*. **(A)** Transcript levels of *GhSWEET42* in wild-type and *GhSWEET42*-overexpressing plants as determined by RT-PCR. *AtUBQ5* was used as an internal control. **(B)** Transcript levels of *GhSWEET42* in wild-type and *GhSWEET42*-overexpressing plants as determined by qRT-PCR. *AtUBQ5* was used as an internal control. Error bars represent standard deviation of three biological replicates. Asterisks indicate significant differences compared with WT. **(C)** Glucose contents in wild-type and *GhSWEET42*-overexpressing plants. Error bars represent standard deviation of three biological replicates. Asterisks indicate significant differences compared with WT. **(D)** Symptoms of wild-type and *GhSWEET42*-overexpressing plants inoculated with *V. dahliae*. Two-week-old *A. thaliana* plants were inoculated with *V. dahliae* and replanted in soil. Plants were photographed at 7 day after inoculation. Analysis was completed using at least 20 plants per line. **(E)** Fungal biomass in wild-type and *GhSWEET42*-overexpressing plants as determined by qRT-PCR. Error bars represent standard deviation of three biological replicates. Asterisks indicate significant differences compared with WT. **(F)** Disease indices of wild-type and *GhSWEET42*-overexpressing plants. Asterisks indicate significant differences compared with WT. Data were generated for three replicates, each comprising 20 *A. thaliana* plants. Data were analyzed using Student’s *t*-test (^∗∗^*P* < 0.01). WT: wild-type plants; OE: GhSWEET42-overexpressing plants.

Transgenic *A. thaliana* lines expressing *GhSWEET42* were generated to characterize its function. The expression of *GhSWEET42* in the T_3_ homozygous transgenic lines was evaluated by RT-PCR and qRT-PCR. Three independent transgenic lines with high *GhSWEET42* expression levels (Figures 5A,B) were chosen for further analyses. When grown under the same conditions, the transgenic plants accumulated more glucose than did wild-type plants (Figure 5C), indicating that the overexpression of *GhSWEET42* affects glucose metabolism in *A. thaliana*. The transgenic and wild-type plants were also compared in terms of their responses to infection by *V. dahliae* strain V592. The inoculated transgenic plants showed more severe disease symptoms (e.g., wilting, chlorosis, early senescence, and necrosis) than did the wild-type plants (Figure 5D). Moreover, the qRT-PCR data confirmed that the fungal biomass was higher in transgenic plants than in wild-type plants (Figure 5E). The disease index calculated from at least 20 plants per treatment was approximately 32 for wild-type plants, but significantly higher for the three transgenic lines overexpressing *GhSWEET42* (i.e., 58, 55, and 51) (Figure 5F). These results indicate that overexpression of *GhSWEET42* increases the glucose content in *A. thaliana* and its sensitivity to *V. dahliae*.

### Down-Regulation of *GhSWEET42* in Cotton Decreases the Glucose Content and Enhances Resistance to *V. dahliae*

**FIGURE 6:**
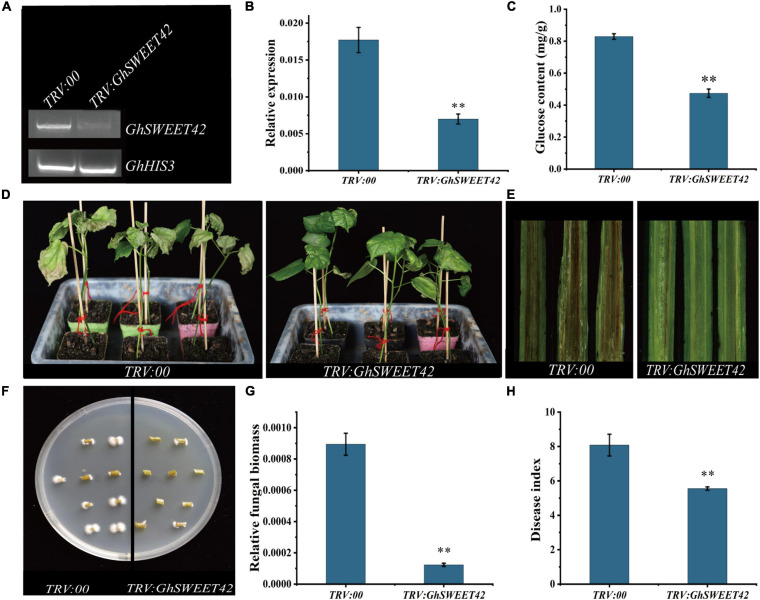
Increased *Verticillium dahliae* resistance of *GhSWEET42*-silenced cotton plants. **(A)** RT-PCR analysis confirming gene-silencing efficiency in roots of *TRV:00* and *TRV:GhSWEET42* plants. *GhHIS3* served as the internal reference control. **(B)** qRT-PCR analysis confirming gene-silencing efficiency in roots of *TRV:00* and *TRV:GhSWEET42* plants. *GhHIS3* served as the internal reference control. Error bars represent standard deviation of three biological replicates. Asterisks indicate significant differences compared with *TRV:00*. **(C)** Glucose contents in roots of *TRV:00* and *TRV:GhSWEET42* plants. Error bars represent standard deviation of three biological replicates. Asterisks indicate significant differences compared with *TRV:00*. **(D)** Representative images of *TRV:00* and *TRV:GhSWEET42* plants infected with *V. dahliae*. After the positive seedlings were completely albino, both plant lines were inoculated with *V. dahliae*. Each biological repeat consisted of at least 30 seedlings. **(E)** Vascular tissues of *TRV:00* and *TRV:GhSWEET42* plants infected with *Verticillium dahliae*. Samples were examined using a stereo microscope. **(F)** Fungal recovery experiments. Stem sections of *TRV:00* and *TRV:GhSWEET42* plants were placed on potato dextrose agar medium and incubated at 25°C. Samples were photographed 3 day later. **(G)** Fungal biomass in *TRV:00* and *TRV:GhSWEET42* plants as determined by qRT-PCR. Error bars represent standard deviation of three biological replicates. Asterisks indicate significant differences compared with *TRV:00*. **(H)** Disease indices of *TRV:00* and *TRV:GhSWEET42* plants. Data were generated for three replicates, each comprising 30 cotton plants. Asterisks indicate significant differences compared with *TRV:00*.Data were analyzed using Student’s *t*-test (^∗∗^*P* < 0.01).

To elucidate the role of *GhSWEET42* during the infection of cotton by *V. dahliae*, *GhSWEET42* expression was knocked-down using a VIGS method. After the plants injected with *TRV:GhCLA1* exhibited the strong albino phenotype (Supplementary Figure 5), the roots from cotton seedlings injected with *TRV:00* and *TRV:GhSWEET42* were sampled for RT-PCR and qRT-PCR analyses. The results confirmed that the transcript level of *GhSWEET42* was lower in the *TRV:GhSWEET42* plants than in the *TRV:00* plants (Figures 6A,B). And the transcript level of *GhSWEET40*, which have a close phylogenetic relationship to *GhSWEET42* (Figure 2A), did not differ significantly between *TRV:GhSWEET42* and *TRV:00* plants (Supplementary Figure 6), showing that *GhSWEET42* was efficiently and specifically silenced in the *TRV:GhSWEET42* plants. Compared with the *TRV:00* plants, the *TRV:GhSWEET42* plants showed significantly decreased glucose content in the roots (Figure 6C). Moreover, the *TRV:GhSWEET42* plants were more resistant to *V. dahliae* than were the *TRV:00* plants, with less wilting and fewer etiolated and abscised leaves (Figure 6D). A stereo microscope was used to examine the accumulation of *V. dahliae* in vascular tissues. And the results showed that the *TRV:GhSWEET42* plants were less affected by *V. dahliae* than were the *TRV:00* plants (Figure 6E). We conducted a recovery assay to assess the extent of the colonization of stems by *V. dahliae*, and found that there was substantially less fungal growth from the stems of *TRV:GhSWEET42* plants than from the stems of *TRV:00* plants (Figure 6F). This result was consistent with the results of the qRT-PCR analysis of fungal biomass (Figure 6G). Finally, we calculated the disease index from at least 30 plants per treatment. The disease index of *TRV:00* plants (83.8) was higher than that of the *TRV:GhSWEET42* plants (55.5) (Figure 6H). These observations suggest that silencing *GhSWEET42* can decrease the glucose level in cotton plants, thereby increasing their resistance to *V. dahliae*.

## Discussion

*Verticillium dahliae* is a soil-borne fungus that attacks plants through the roots (Fradin and Thomma, 2006). *V. dahliae* needs to penetrate the root epidermal cells to complete the colonization process, and this stage is critical for successful infection by *V. dahliae* (Bowers et al., 1996). Since marker-expressing fungal strains can be used to analyze the fungal colonization process, GFP transformants have become a common tool for analyzing various colonization processes of fungi in plants (Oren et al., 2003; Pantelides et al., 2009). In this study, cotton roots were infected with V592-GFP to observe the colonization process of *V. dahliae*. The results identified 48 hpi as a critical period for the colonization of cotton roots by *V. dahliae*. Another study also found that 48 h is a critical period for *V. dahliae* to penetrate epidermal cells, and the hyphae swell slightly to form penetrating structures when penetrating the epidermal cells of *A. thaliana* (Zhao et al., 2014). After successful penetration into the epidermal cells, hyphae grow and branch within the root cortex and begin to form networks (Zhang et al., 2012). In the study, the observation showed that many hyphae appeared and colonized the root cells at 96 hpi.

Numerous studies have found that sugars play a central role in the host-pathogen interaction (Chen et al., 2010; Gupta, 2020). Sugar transport is crucial for the pathogenicity of the fungus (Doidy et al., 2012). Pathogen infection triggers changes in sugar transport in host plants to improve nutrient supply to the pathogen (Lemoine et al., 2013). For example, previous studies have shown that sucrose and hexose significantly accumulate in *Pst*-challenged wheat leaves (Chang et al., 2013), and the glucose content increases in adult leaves of *Zea mays* infected with *U. maydis* (Sosso et al., 2019). A study on nutrient transfer from the host to the fungus found that glucose is the main carbon and energy source transferred to the fungal hyphae (Sutton and Hall, 1999). The increased sugar contents in infected tissues may be due to increased import of materials from adjacent uninfected tissues and healthy leaves (Chou et al., 2000; Hayes et al., 2010). We found that the glucose content was significantly increased at 48 hpi in the cotton roots infected by *V. dahliae*, suggesting that *V. dahliae* infection might result in glucose transport from uninfected cells to infected cells in cotton.

Disease resistance in plants is generally controlled by dominant resistance (*R*) genes. In a few cases, the gene that provides resistance is recessive, so plants carrying the corresponding dominant allele are sensitive (Navathe et al., 2020). In these cases, the dominant allele is a susceptibility (*S*) gene. The *SWEET* family, which is involved in sucrose or glucose transport, is among the most intensively studied class of *S* genes (Chen et al., 2010; Yuan and Wang, 2013). These sugar transporters are often hijacked by pathogens to supply sugars as a source of nutrition for themselves, leading to successful infection (Chen et al., 2010). Infection by pathogens leads to up-regulation of clade II SWEET genes in *A. thaliana* and *V. vinifera* (Chen et al., 2010; Chong et al., 2014). We found that the infection by *V. dahliae* up-regulated the clade II SWEET gene *GhSWEET42*. And overexpression of *GhSWEET42* in *A. thaliana* increased the glucose accumulation, and enhanced susceptibility to *V. dahlia*. Previous studies showed that pathogens generally attack the host by secreting transcription activator-like effectors (TALEs), which enter the nuclei of host cells and mimic transcription factors to increase the expression of SWEET genes (Cox et al., 2017; Deng et al., 2017). For example, PthXo1 secreted by *Xanthomonas oryzae* pv. *Oryzae* (*Xoo*) strain PXO99^A^ is a TALE, which directly interacts with the *OsSWEET11* promoter and specifically activates the transcription of *OsSWEET11* in rice (Yang et al., 2006). Another study found that Avrb6, a TALE determining *Xanthomonas citri* subsp. *malvacearum* pathogenicity, upregulates *GhSWEET10* expression in cotton (Cox et al., 2017). The increased expression of sugar transporters stimulated by pathogenic bacteria would divert host nutrition toward infection sites (Walerowski et al., 2018). Therefore, GhSWEET42 sugar transporter might be the target of *V. dahliae*, and may facilitate glucose accumulation at the site of infection to enhance *V. dahliae* growth.

In many cases, the pathogenicity of pathogens depends on the sugar supply by the sugar transporters encoded by SWEET genes of the hosts. Therefore, modulating the host SWEET genes to block pathogen access to nutrients has emerged as an attractive strategy to achieve disease resistance (Oliva and Quibod, 2017). In our study, silencing *GhSWEET42* expression in cotton decreased the glucose content and improved resistance to *V. dahliae*. Previous studies demonstrated that the *A. thaliana sweet4* mutant shows enhanced resistance to gray mold (Chong et al., 2014), and inhibiting *OsSWEET11* function in mesophyll cells increases the resistance of rice to sheath blight disease (Gao et al., 2018). Additionally, altering the TALE binding elements in *SWEET* gene promoters by CRISPR/Cas9-mediated genome editing endows rice lines with robust, broad-spectrum resistance to bacterial blight (Eom et al., 2019; Oliva et al., 2019). Thus, the identification of *GhSWEET42* provides a candidate gene for using the sugar starvation strategy to inhibit infection by *V. dahliae* in cotton.

In conclusion, the results of this study revealed 48 hpi as a key period during the colonization of cotton roots by *V. dahliae*. Additionally, we proved that *V. dahliae* infection increased the transport of glucose to the infection site. We observed that the ectopic expression of *GhSWEET42* in *A. thaliana* increased the glucose content in the transformed plants. During infection of these transgenic *A. thaliana* plants by *V. dahliae*, the pathogen grew quickly, leading to accelerated development of disease symptoms. Conversely, silencing of *GhSWEET42* decreased the glucose content in roots and reduced disease symptoms and restricted hyphal growth when infected by *V. dahliae*. Therefore, *GhSWEET42* was involved in infection of *V. dahliae* in cotton through glucose translocation and could be as a target gene to strengthen cotton resistance to *V. dahliae*.

## Data Availability Statement

The original contributions presented in the study are included in the article/Supplementary Material, further inquiries can be directed to the corresponding authors.

## Author Contributions

WL, XM, and DY conceived and designed the research. MS, ZZ, ZR, XW, and JZ performed the experiments. WS, HF, and FZ provided the materials. MS, ZZ, WL, WS, and HF analyzed the data. MS and WL prepared the figures and wrote the manuscript. XM and DY revised the manuscript. All authors have read and approved the final manuscript.

## Conflict of Interest

The authors declare that the research was conducted in the absence of any commercial or financial relationships that could be construed as a potential conflict of interest.

## Publisher’s Note

All claims expressed in this article are solely those of the authors and do not necessarily represent those of their affiliated organizations, or those of the publisher, the editors and the reviewers. Any product that may be evaluated in this article, or claim that may be made by its manufacturer, is not guaranteed or endorsed by the publisher.

## Supplementary Material

Supplementary Figure 1 Fluorescence images at 0, 6, 12, and 24 h after infection by *Verticillium dahliae*.

Supplementary Figure 2 Bright field images at 48 and 96 h after infection by *Verticillium dahliae*.

Supplementary Figure 3 Relative transcript levels of *GhSWEET42* in roots of resistant and susceptible cotton varieties at 48 h after infection by *Verticillium dahliae*. The relative transcript levels of *GhSWEET42* were detected by qRT-PCR, and *GhHIS3* served as the internal reference control. Resistant variety: Zhongzhimian 2; susceptible variety: TM-1. Asterisks indicate significant differences compared with Zhongzhimian 2. Error bars represent standard deviation of three biological replicates. Data were analyzed using Student’s *t*-test (***P* < 0.01).

Supplementary Figure 4 Multiple sequence alignment and transmembrane structural analysis of GhWEET42.

Supplementary Figure 5 Albino phenotype of *TRV:GhCLA1* plants.

Supplementary Figure 6 The expression analysis of *GhSWEET40* in roots of *TRV:00* and *TRV:GhSWEET42* plants by qRT-PCR. *GhHIS3* served as the internal reference control. Error bars represent standard deviation of three biological replicates. n.s. indicates no significant difference compared with *TRV:00*. Data were analyzed using Student’s *t*-test (***P* < 0.01).

Supplementary Table 1 Protein sequences of *SWEET* genes from *Gossypium hirsutum*, *Arabidopsis thaliana*, and *Vitis vinifera*.

Supplementary Table 2 Primers used in this study.

Supplementary Table 3 Association analysis of natural variations around *SWEET* genes with *Verticillium dahliae* resistance in cotton lines and cultivars.
