# Supplementary figures and images for: The GhSWEET42 Glucose Transporter Participates in Verticillium dahliae Infection in Cotton
Source: Front Plant Sci. 2021 Jul 27;12:690754. doi: 10.3389/fpls.2021.690754 (PMC8353158; doi:10.3389/fpls.2021.690754)

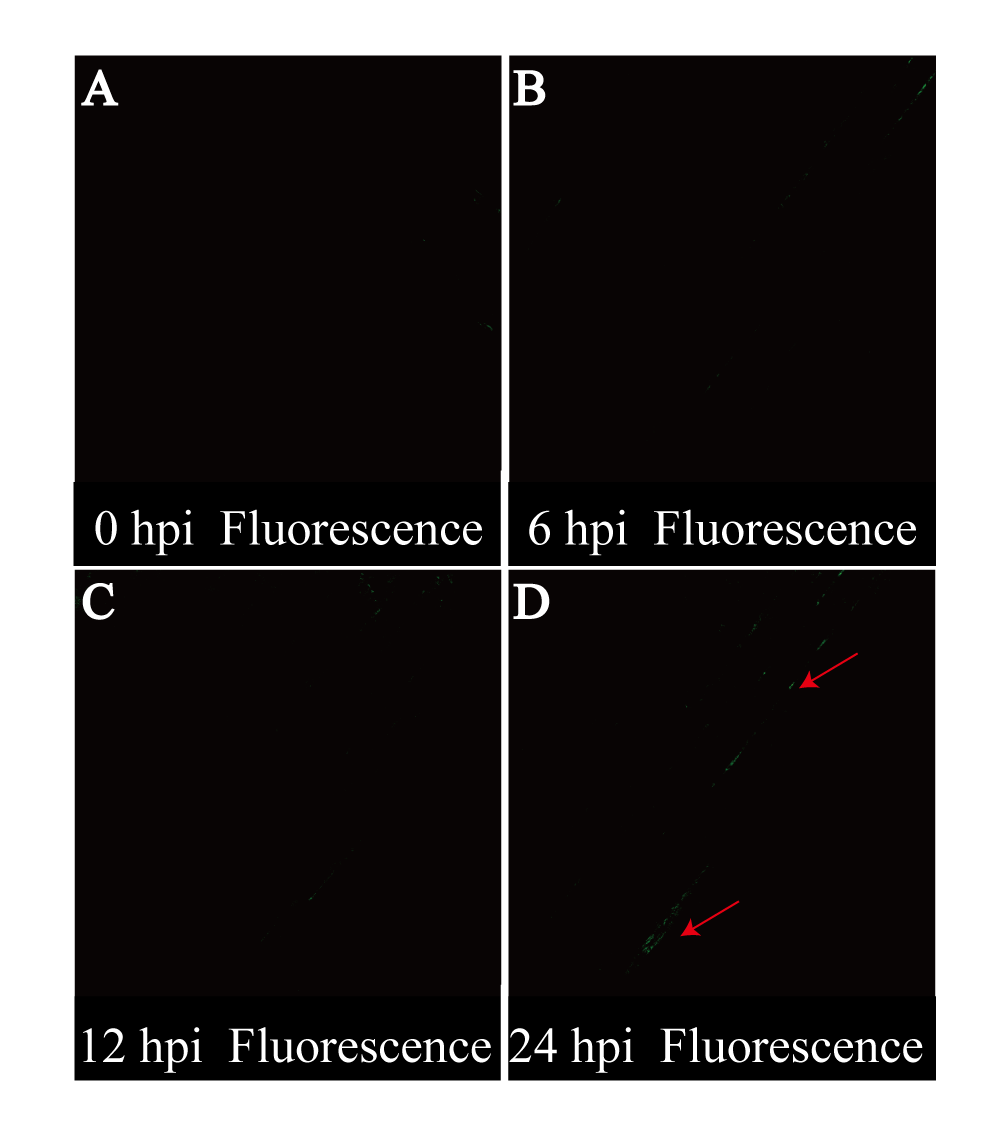

Supplement: Supplementary Figure 1 — Fluorescence images at 0, 6, 12, and 24 h after infection by Verticillium dahliae. [file Image_1.TIF]

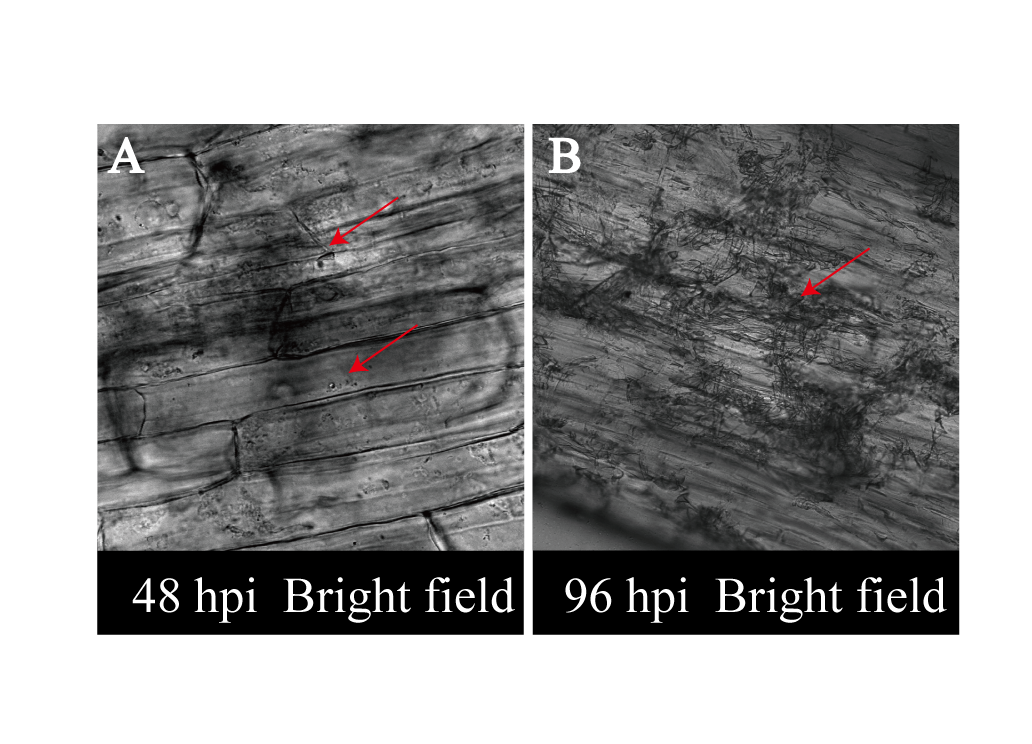

Supplement: Supplementary Figure 2 — Bright field images at 48 and 96 h after infection by Verticillium dahliae. [file Image_2.TIF]

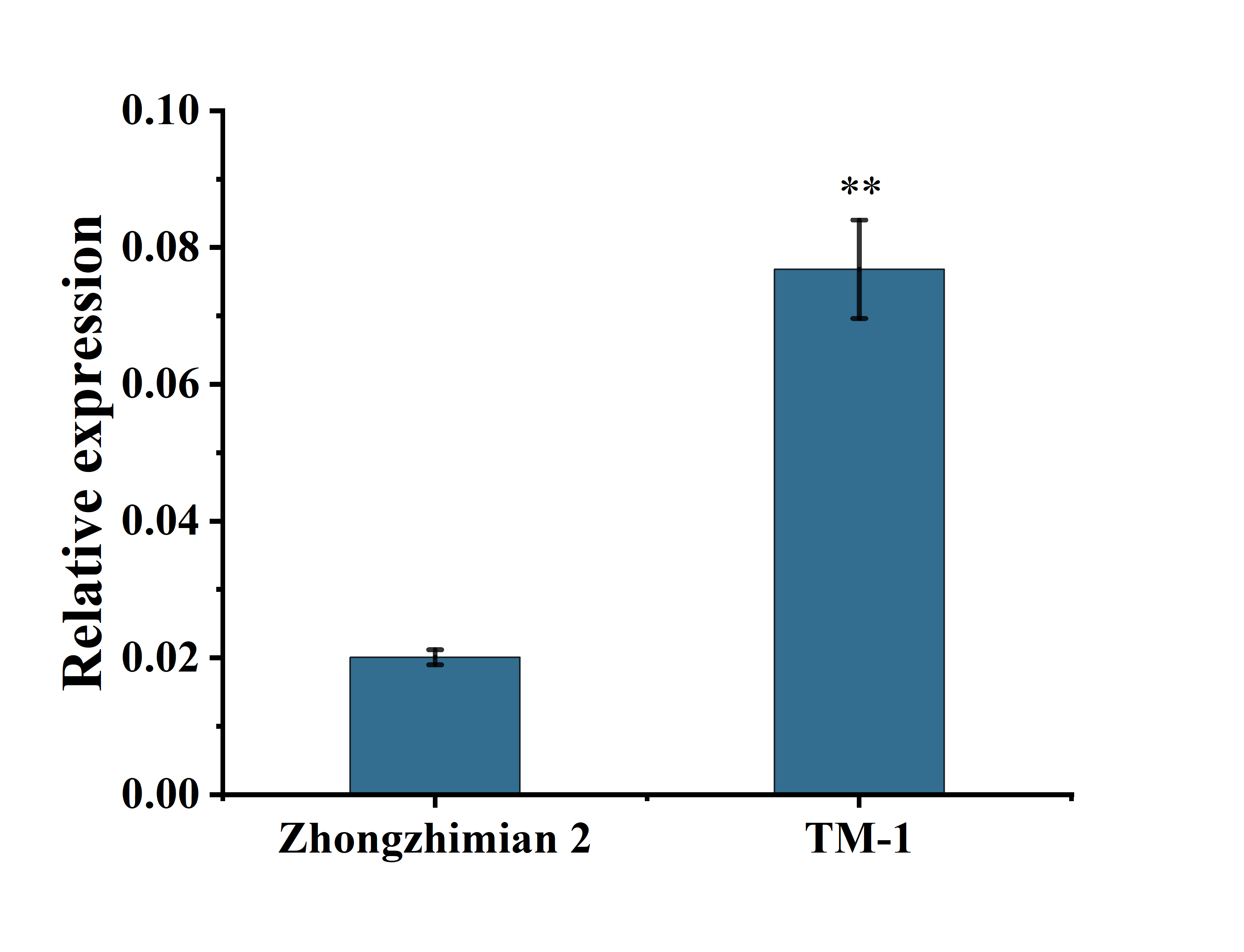

Supplement: Supplementary Figure 3 — Relative transcript levels of GhSWEET42 in roots of resistant and susceptible cotton varieties at 48 h after infection by Verticillium dahliae. The relative transcript levels of GhSWEET42 were detected by qRT-PCR, and GhHIS3 served as the internal reference control. Resistant variety: Zhongzhimian 2; susceptible variety: TM-1. Asterisks indicate significant differences compared with Zhongzhimian 2. Error bars represent standard deviation of three biological replicates. Data were analyzed using Student’s t-test (**P < 0.01). [file Image_3.TIF]

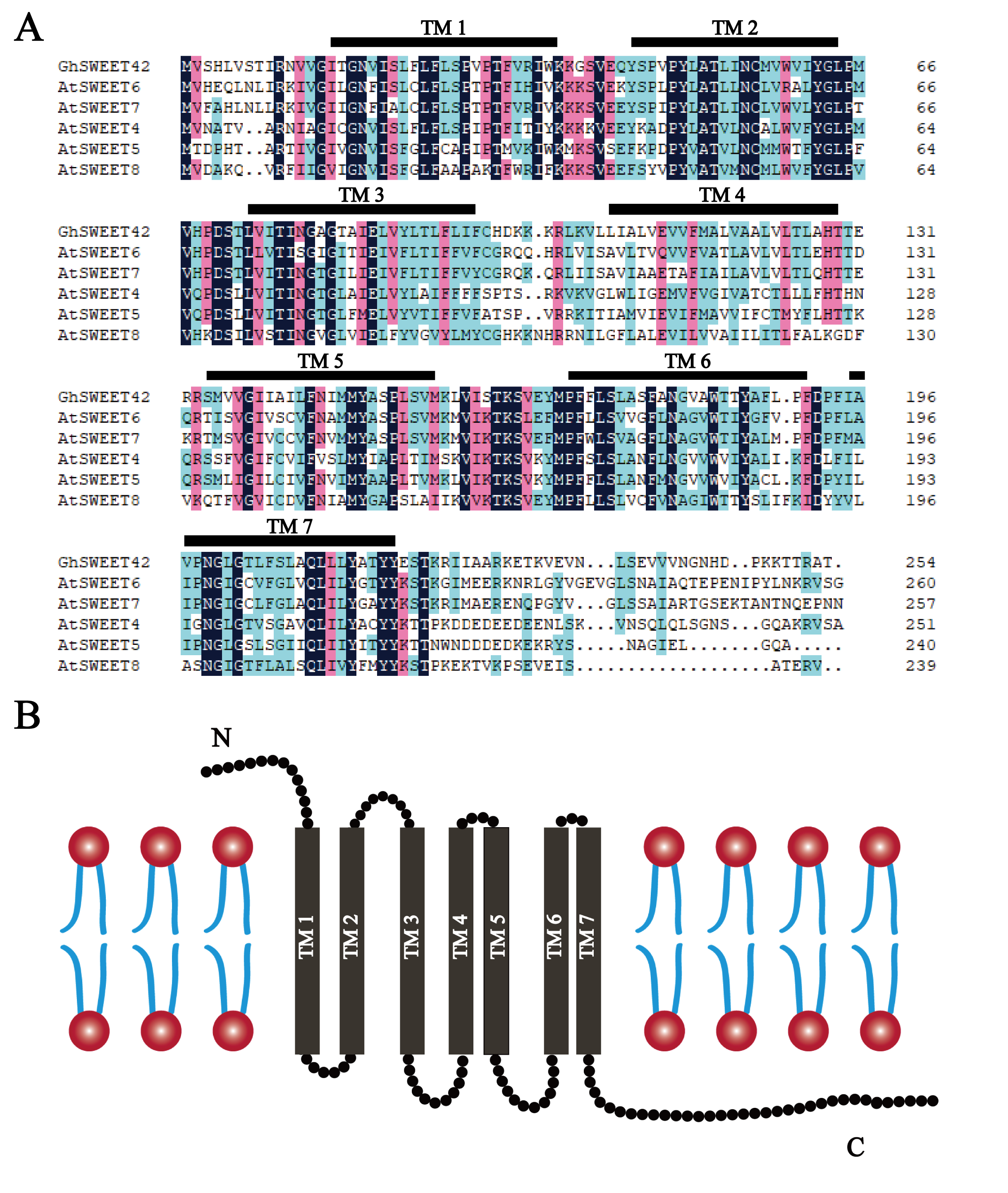

Supplement: Supplementary Figure 4 — Multiple sequence alignment and transmembrane structural analysis of GhWEET42. [file Image_4.TIF]

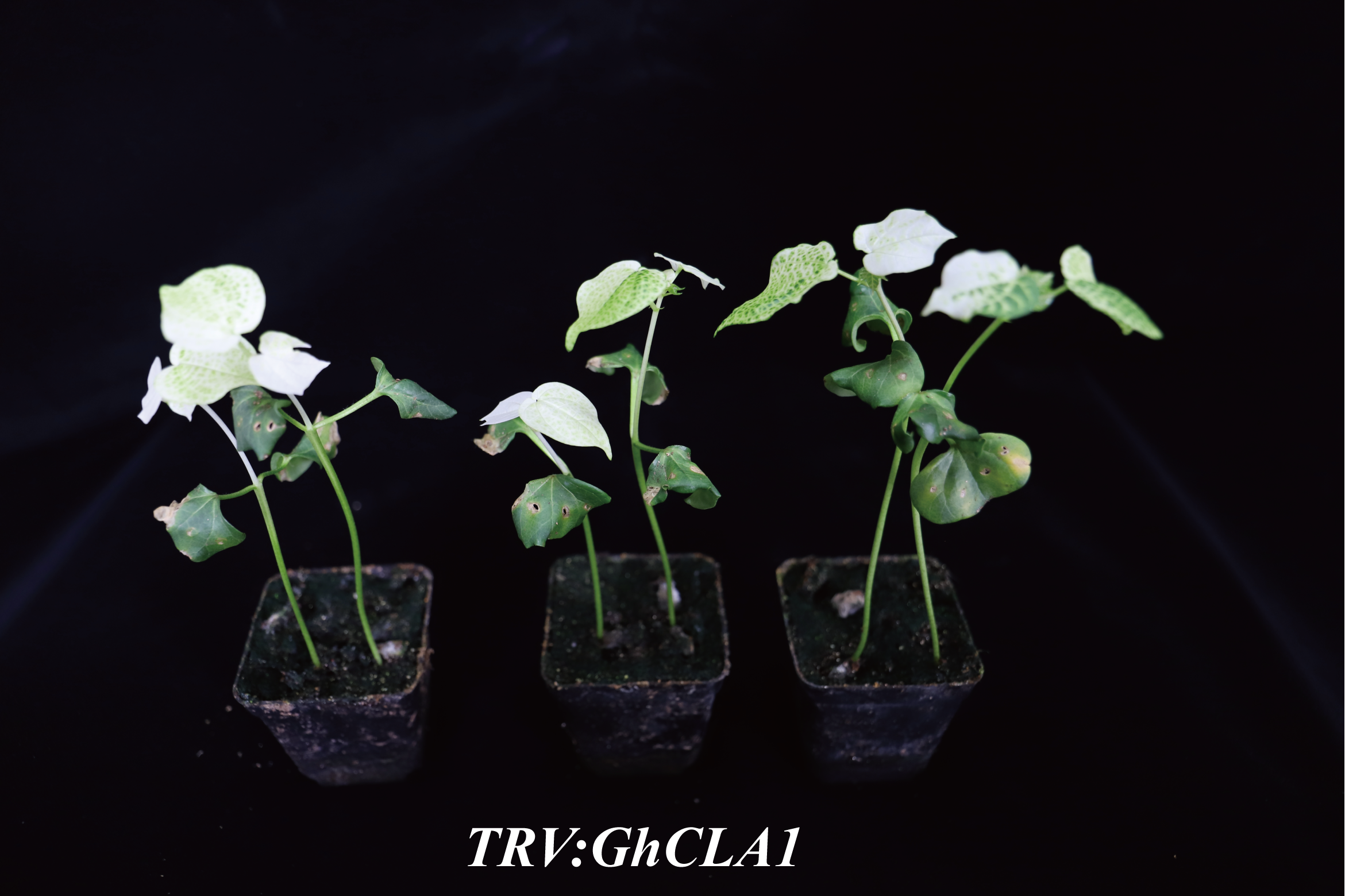

Supplement: Supplementary Figure 5 — Albino phenotype of TRV:GhCLA1 plants. [file Image_5.TIF]

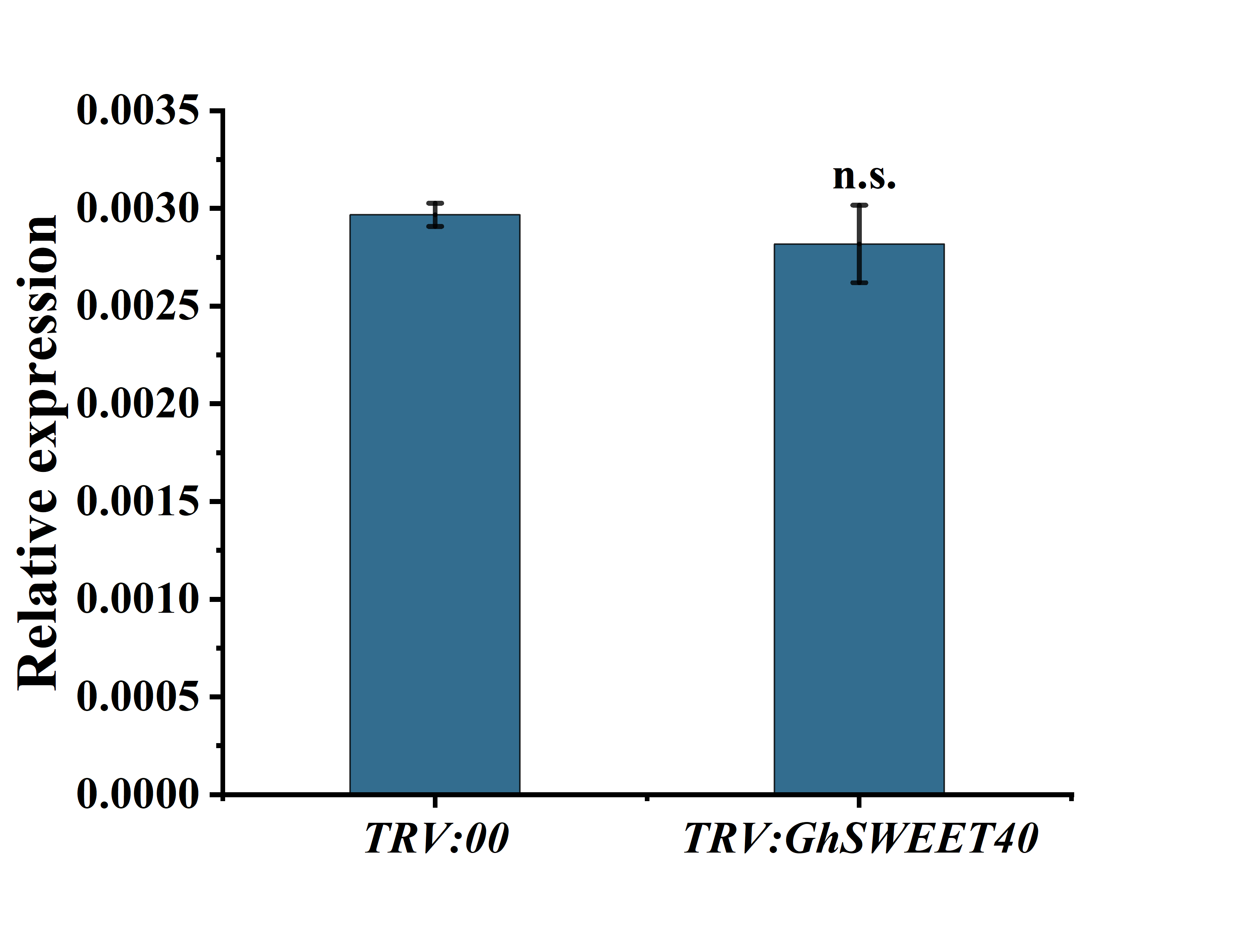

Supplement: Supplementary Figure 6 — The expression analysis of GhSWEET40 in roots of TRV:00 and TRV:GhSWEET42 plants by qRT-PCR. GhHIS3 served as the internal reference control. Error bars represent standard deviation of three biological replicates. n.s. indicates no significant difference compared with TRV:00. Data were analyzed using Student’s t-test (**P < 0.01). [file Image_6.TIF]
